# Research on image content description in Chinese based on fusion of image global and local features

**DOI:** 10.1371/journal.pone.0271322

**Published:** 2022-08-29

**Authors:** Dongyi Kong, Hong Zhao, Xiangyan Zeng

**Affiliations:** 1 School of Computer and Communication, Lanzhou University of Technology, Lanzhou, China; 2 Department of Mathematics and Computer Science, Fort Valley State University, Fort Valley, GA, United States of America; Newcastle University, UNITED KINGDOM

## Abstract

Most image content modelling methods are designed for English description which is different form Chinese in syntax structure. The few existing Chinese image description models do not fully integrate the global features and the local features of an image, limiting the capability of the models to represent the details of the image. In this paper, an encoder-decoder architecture based on the fusion of global and local features is used to describe the Chinese image content. In the encoding stage, the global and local features of the image are extracted by the Convolutional Neural Network (CNN) and the target detection network, and fed to the feature fusion module. In the decoding stage, an image feature attention mechanism is used to calculate the weights of word vectors, and a new gating mechanism is added to the traditional Long Short-Term Memory (LSTM) network to emphasize the fused image features, and the corresponding word vectors. In the description generation stage, the beam search algorithm is used to optimize the word vector generation process. The integration of global and local features of the image is strengthened to allow the model to fully understand the details of the image through the above three stages. The experimental results show that the model improves the quality of Chinese description of image content. Compared with the baseline model, the score of CIDEr evaluation index improves by 20.07%, and other evaluation indices also improve significantly.

## 1. Introduction

Image content description, also known as image semantic understanding, uses computer vision and deep learning technology to extract the semantics contained in the image, and use natural language processing technology to generate a reasonable text description [1, 2]. Image content description belongs to the task of cross modal transformation, which is different from image classification and object detection task of computer vision. Image semantic understanding includes extracting the semantic information in an image and converting it into fluent text descriptions. Image content description breaks through the barriers between computer vision and natural language processing, and organically integrates the two directions in the field of artificial intelligence. The challenge is to accurately transform the image features obtained by computer vision into the text information generated by natural language processing.

With the wide application of deep learning, convolutional neural network has been used in the field of image content description. Through convolutional neural network, the model can extract image features independently to understand the semantic information contained in the image, and get better results in the task of image content description. However, there are some problems in the current work. First, most of the existing researches either use convolution neural network to extract global image features [3–8] or use Fast R-CNN [9] and other target detection networks to recognize targeted local objects in the image [10–12]. They do not integrate the global features and the local features, which limits the improvement of the model description performance. Secondly, in the descriptive statement generation stage of the existing models [7, 13–15], the word vector with the highest probability among the candidate words is chosen by the model as the final word and output it directly as a sentence. However, the sentence obtained in this way is not necessarily the best descriptive result. Thirdly, most of the current research work focus on English image description [5–8, 10], There are only a few researches on image content description in Chinese [4, 11, 16].

We propose a Chinese image content description method based on the fusion of global and local features. The main contributions are as follows: (1) In the encoding stage, the global and local features of the image are first extracted respectively. Then, the feature fusion module is constructed by using convolution kernel, Multilayer Perceptron (MLP) and other technologies to fuse the global and local features. (2) In the decoding stage, attention weights are calculated for image features and a new gate unit is added to the LSTM network to adjust image fusion features with attention information, where the model can better focus on the global and local features in the image fusion features to improve the decoding ability of the model. (3) in the description statement generation stage, beam search [17] is used to search and combine the candidate word vectors generated by the model to optimize the description generation results and further improve the model description generation effect.

## 2. Related work

### 2.1 Encoder-decoder structure

Mao et al. [18] proposed a multimodal Recurrent Neural Networks (m-RNN) model. In this model, the encoder decoder structure is used in the field of image content description for the first time. A convolutional neural network is used to encode the image, and a Recurrent Neural Network (RNN) [19] is used to decode the extracted features, realizing cross modal fusion between image feature information and text description information. Vinyals et al. [6] proposed the Neural Image Caption (NIC) model, which replaces the decoder in the m-RNN model with LSTM [20], which makes the model have strong long-term memory ability and improves the description performance of the model. Wu et al. [21] constructed a large-scale ICC dataset with the most comprehensive scenes and the richest language description. The dataset contains 300000 images and 1.5 million Chinese description sentences. The NIC model is used to verify the dataset, and the results show that the dataset effectively improves the performance of the existing models.

### 2.2 Attention mechanism

Xu et al. [7] proposed an image description method with a feature attention mechanism inspired by work in machine translation. It calculates the weights of words in the description text and generates an image feature vector with the weight information. The decoding network can adjust image features with different weights at different times, which can enhance image features and alleviate overfitting. Lu et al. [8] proposed an image description model based on an adaptive attention mechanism, which calculates the importance of image description vocabulary in images by introducing visual sentinels. The visual sentinels decide whether the final predicted vocabulary is generated directly using a language model or by using the attention mechanism to calculate the attention weights of the word vectors. This assigns greater weight to the more important features in the image. Liu et al. [4] proposed a Chinese image content description model based on via visual attention [22] and topic modeling, which reduces the bias between image semantics and description statements by adding visual attention mechanism, and improves the accuracy of description statement generation by extracting the theme features of images through theme modeling. Zhao et al. [23] proposed a Chinese description method of image content based on image feature attention and adaptive attention fusion. The two attention mechanisms of literature [7] and literature [8] are fused in depth to extract more accurate information about the main features in the image, which effectively improves the image understanding and description capability of the model.

### 2.3 Local image features

Anderson et al. [10] proposed a Bottom-Up and Top-Down (BUTD) attention model, in which Faster R-CNN was used to extract local features in the image and a bottom-up attention mechanism was used to identify the image feature areas, and then a top-down attention mechanism was used to determine the weight values of image features. Ma et al. [11] proposed an improved Chinese image description model based on a global attention mechanism. This model adds global image features to the BUTD, which effectively overcomes the semantic loss caused by the loss of global features. However, this model does not deeply fuse global and local features. Li and Chen [14] proposed an image description model based on the fusion of image local features and label attributes. The model uses the target detection method and attribute trainer to extract the local features of the image and the Attributes as high-level semantic of the image features, and decodes the two features after fusion. Zhang et al. [12] used Faster R-CNN to extract local image features, used visual semantic attention model to generate visual keywords, and added optimized pointer network to the model, so that the model can receive variable length input sequence.

The above methods [3–8, 10–12, 23] obtain either global or local features, which will lead to incomplete image semantic features. Only [11, 14] combined global and local features, but did not deeply integrate the two image features, resulting in poor image content description effect.

## 3. Chinese image description model

### 3.1 Model design

**Fig 1:**
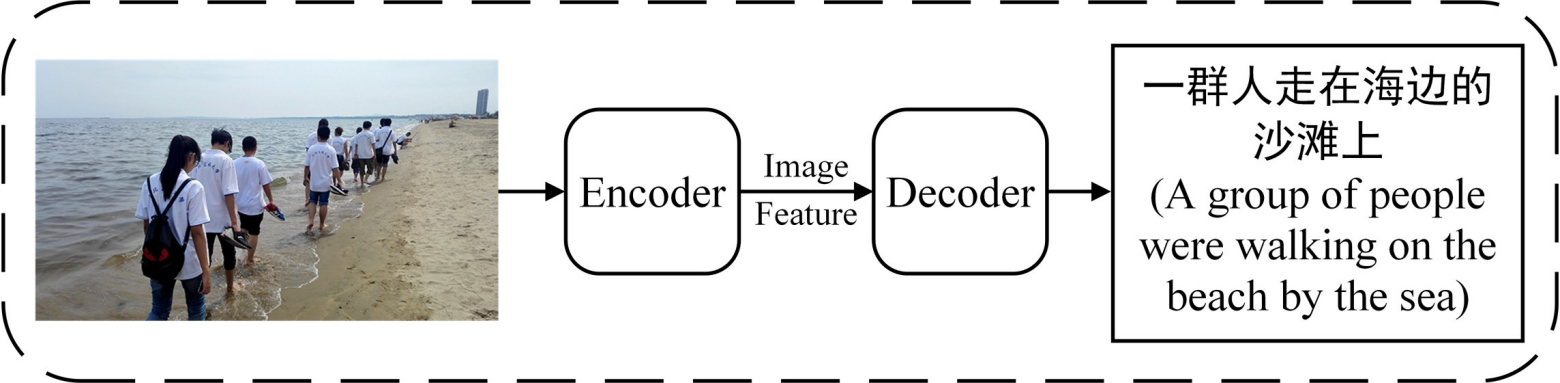
Encoding and decoding structure of Chinese description of image content.

Based on the encoder decoder framework, this research constructs a Chinese image description model with global and local image feature fusion. The encoder decoder structure was first proposed by Cho et al. [24], also known as sequence to sequence (seq2seq) structure [25], which is a model structure in deep learning, as shown in Fig 1.

Mao et al. [18] introduced this structure into the field of image content description. The encoder encodes the input information into an intermediate semantic vector, and the decoder decodes the semantic vector to get the output result. The two parts are independent but closely related to each other, which is conducive to the conversion between different modes. The encoding and decoding process is as follows:

$$V_{C}=\mathrm{Encoder}(x_{1},x_{2},\ldots ,x_{m})$$ (1)

$$y_{t}=\mathrm{Decoder}(V_{C};y_{1},y_{2},\ldots ,y_{t-1})$$ (2)

Where *x*_1_,*x*_2_,…,*x*_*m*_ is the input sequence of the encoder decoder structure, *V*_*C*_ is the semantic vector generated by the encoder, and *y*_*t*_ is the output value of the decoder at *t* time.

### 3.2 Encoding stage

**Fig 2:**
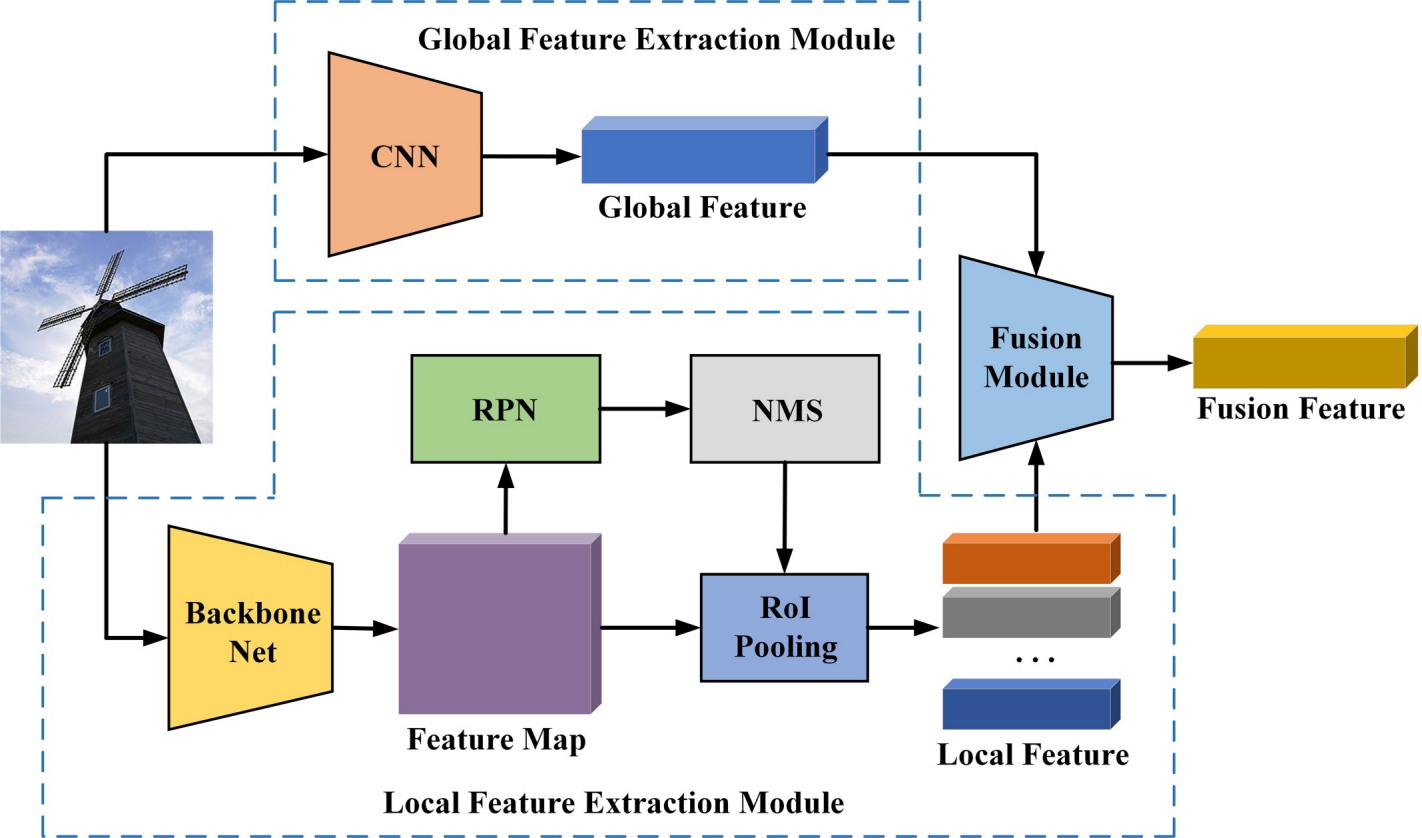
Extraction process of global and local image features.

The model’s extraction of image features is divided into two modules: global feature extraction and local feature extraction. The global image feature extraction module uses CNN to extract the global features of the image. The local image feature extraction module uses the backbone network to extract the underlying features of the image, and then uses the image Region Proposal Network (RPN) to search the local objects and get their coordinate information. Finally, the Region of Interest (RoI) pooling network is used to extract the local features that represent the image details from the underlying features through feature mapping. The fusion of global features and local features generates represents image content, as shown in Fig 2.

#### 3.2.1 Global image feature extraction

The global image feature extraction module is a pre-trained ResNet152 [26], where the average pooling layer and the fully connected layer of 1*1 are replaced by the average pooling layer of 14*14. The global image feature vector *V*_*g*_ is extracted by the network:

$$V_{g}=\{v_{1},v_{2},\ldots ,v_{i},\ldots ,v_{n}\}$$ (3)

Where *v*_*i*_∈R^*M*^ is the image feature at any position in the image feature vector, and *M* is the dimension size of the image feature; *n* is the number of image features.

#### 3.2.2 Local image feature extraction

The common features extracted by the model through ResNet50 are shared by the subsequent local candidate region generation network and RoI pooling network to form the underlying image feature *V*_*p*_, as follows:

$$V_{p}=\{v_{1}^{\prime },v_{2}^{\prime },\ldots ,v_{i}^{\prime },\ldots ,v_{k}^{\prime }\}$$ (4)

Where $v_{i}^{\prime }\in R^{N}$ is the image feature at any position in the common feature graph, and *N* is the dimension size of the image feature; *k* is the number of image features.

By using RPN and Non-Maximum Suppression (NMS) [27] algorithm, the local target object is screened out from the image bottom feature *V*_*p*_ and its coordinate information *G* is predicted. Then, according to the coordinate information *G* of the candidate box, the features are extracted from the bottom feature *V*_*p*_ through the mapping relationship, and the image feature *V*_*p*_ of the ROI region is obtained as follows:

$$V_{R}=\mathrm{RoI}(V_{p},G)$$ (5)

Finally, the RoI pooling network is used to extract image feature *V*_*R*_ of the region of interest (ROI) corresponding to the candidate box, and local feature vector *V*_*l*_ with fixed size is obtained as follows:

$$V_{l}=\mathrm{RoI}\;\mathrm{Pooling}(V_{R})$$ (6)

#### 3.2.3 Global and local image feature fusion

**Fig 3:**
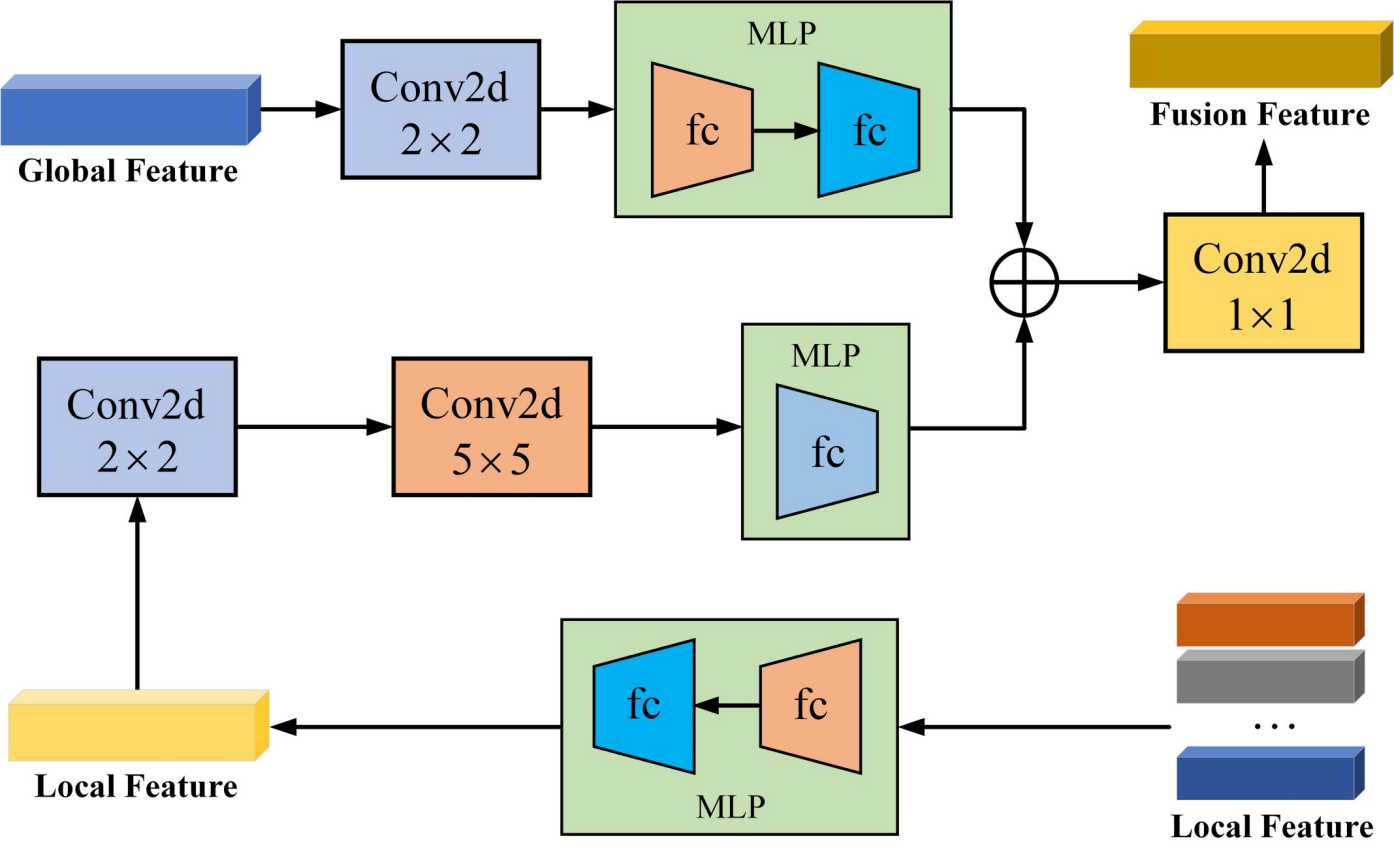
Global and local image feature fusion.

The global and local features of the image are sent to the image feature fusion module, as shown in Fig 3. The feature fusion module consists of three components: global image feature information processing, local image feature information processing and image feature fusion.

In the first component, the global image feature *V*_*g*_ is fed into the convolution layer with convolution kernel size of 2*2 to extract the global feature, which is then sent to a Multilayer Perceptron network. The main functions include: (1) dimensionality reduction on the characteristic vector to reduce the complexity of the model and prevent over fitting; (2) weighting image features to facilitate the subsequent image feature fusion. The calculation process is as follows:

$$V_{a}=\mathrm{MLP}(\underset{2\times 2}{Conv2d}(V_{g}))$$ (7)

Where $\underset{2\times 2}{Conv2d}(∙)$ represents 2D convolution operation with convolution kernel size of 2*2, and MLP(∙) represents Multilayer Perceptron network.

In the second component, the local image feature *V*_*l*_ is passed through a MLP network and two convolution layers with kernel size of 2*2 and 5*5. Compared with the global image feature *V*_*g*_, the local feature *V*_*l*_ has more quantity and more detailed information. Therefore, when processing the local feature *V*_*l*_, the convolution layer with the convolution kernel size of 5*5 is used to extract the feature at a deeper level. Then sent it to the Multilayer Perceptron network for processing. The calculation process is as follows:

$$V_{l}^{\prime }=\mathrm{MLP}(V_{l})$$ (8)

$$V_{b}=\mathrm{MLP}(\underset{5\times 5}{Conv2d}(\underset{2\times 2}{Conv2d}(V_{l}^{\prime })))$$ (9)

Where $\underset{5\times 5}{Conv2d}(∙)$ means to perform 2D convolution operation with convolution kernel size of 5*5.

In the third component, the global image feature *V*_*a*_ and local image feature *V*_*b*_ are fused. Firstly, the matrix addition operation is used to fuse the two features. In the model training, the proportion between the two features can be dynamically adjusted to achieve the best fusion effect. Then the fused features are sent to the convolution layer with convolution kernel of 1*1 for further fusion, and finally the fused image feature *V*_*f*_ is obtained. The calculation process is as follows:

$$V_{f}=\underset{1\times 1}{Conv2d}(V_{a}⊕V_{b})$$ (10)

Where, ⊕ represents matrix addition operation, and $\underset{1\times 1}{Conv2d}(\cdot )$ represents 2D convolution operation with convolution kernel size of 1*1.

### 3.3 Decoding phase

**Fig 4:**
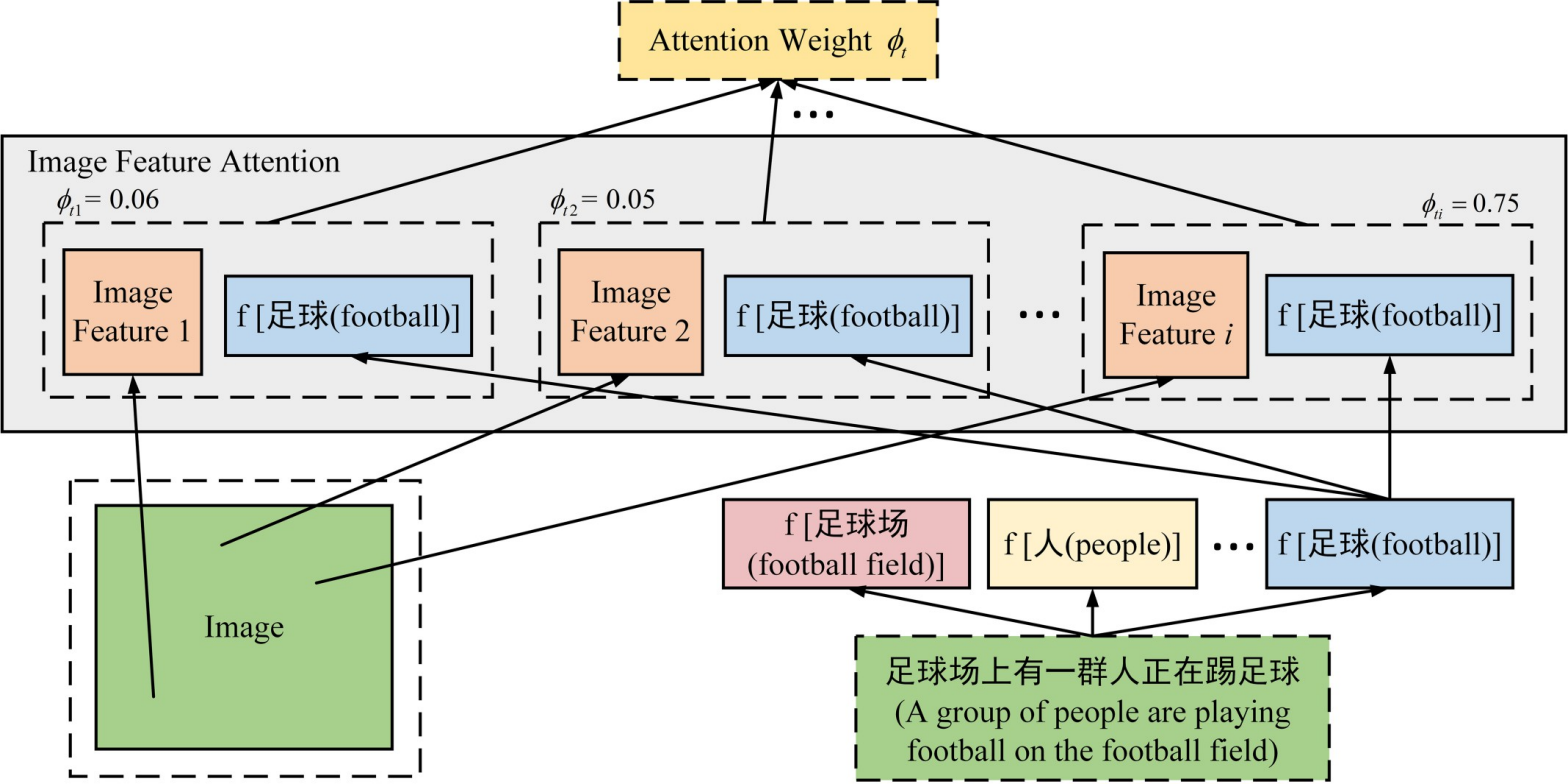
Attention weight of image feature.

**Fig 5:**
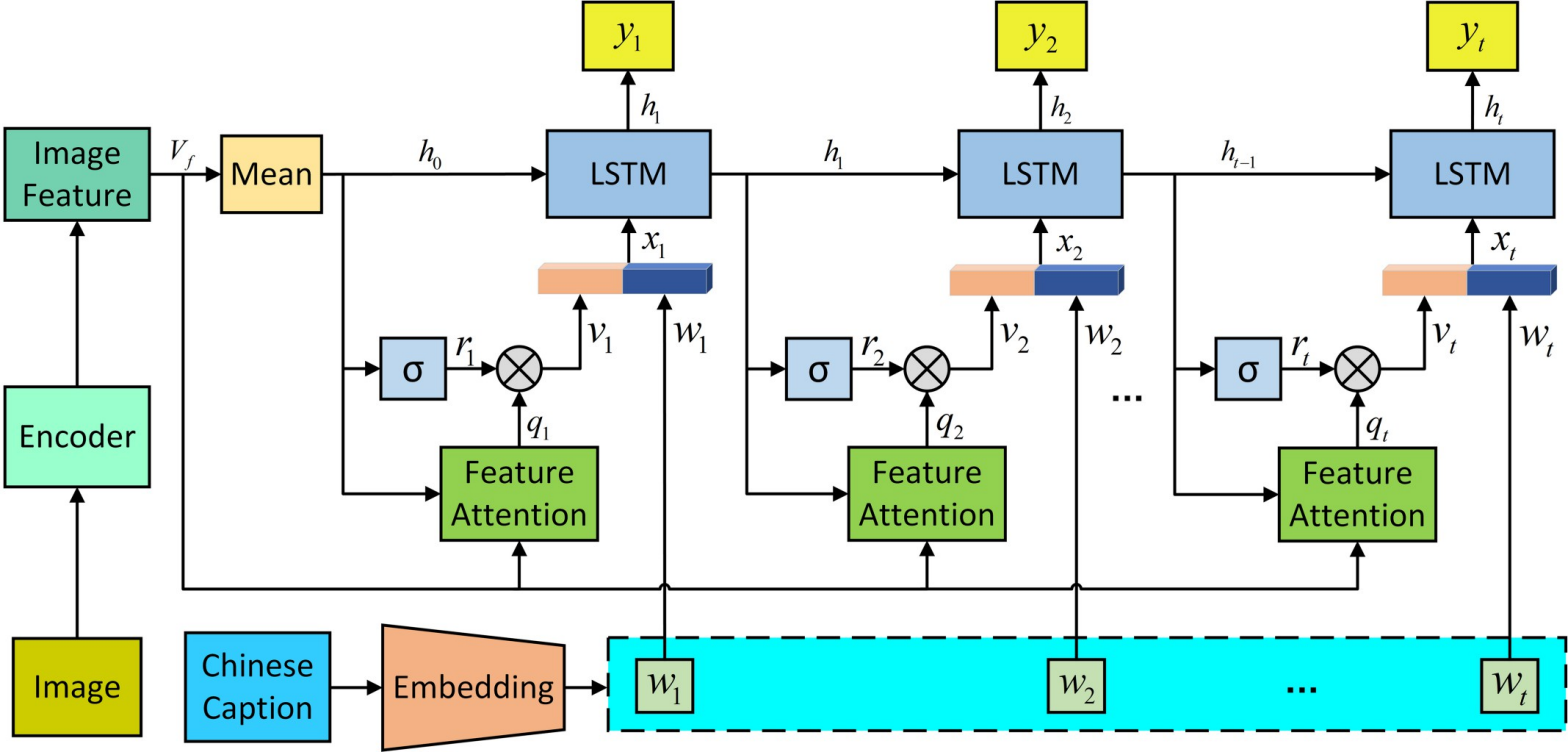
Dynamic adjustment mechanism of image feature attention vector.

In the decoding stage, the model maps the lexical information described in the text of the training set to the corresponding image feature area through the image feature attention mechanism. The calculation process is as follows:

(1) As shown in Fig 4, the attention weight of each region of the image feature at *t* time is calculated. Firstly, MLP is used to couple the image feature *V*_*f*_ with the hidden information *h*_*t*−1_ output by the decoder at the last time. Then, the above calculation results are sent into SoftMax function to calculate the weight value *ϕ*_*ti*_ of the *i*-th image feature region at time *t*, and the weight distribution *ϕ*_*t*_ of each region of the image can be obtained. The sum of the weight distribution is 1, that is ∑_*i*_*ϕ*_*ti*_ = 1. These weight distributions represent the attention degree of the word vector information at time *t* to each region of the image, as follows:
$$d_{ti}=W_{f\_att}(\mathrm{ReLU}(W_{e}\cdot [h_{t-1},V_{f}]+b_{e}))+b_{f\_att}$$ (11)
$$\phi_{ti}=\frac{\exp(d_{ti})}{\sum_{l=1}^{m}\exp(d_{tl})}$$ (12)
  Where *W*_*f*_*att*_, *W*_*e*_, *b*_*f*_*att*_ and *b*_*e*_ are the weight parameters and bias parameters that the Multilayer Perceptron needs to learn, ReLU [28] represents the Rectified Linear Unit, and *m* represents the number of image features.

(2) The attention weight is mapped to the image features. Firstly, the attention model of image features focuses on the target in the image features by threshold *λ*_*t*_. Then, the weight distribution *ϕ*_*ti*_ calculated above is applied to the corresponding image region, and finally the image feature vector *q*_*t*_ with weight information at time *t* is obtained, as follows:
$$\lambda_{t}=\sigma (W_{\beta }h_{t-1})$$ (13)
$$q_{t}=\lambda_{t}\sum_{i=1}^{L}\phi_{ti}v_{i}$$ (14)
  Where *L* is the number of image feature regions and *W*_*β*_ is the weight parameter that threshold *λ*_*t*_ needs to learn.
(3) The generated image feature attention vector is adjusted dynamically. As shown in Fig 5, A new gate unit *r*_*t*_ is added to the traditional LSTM network as follows:

$$r_{t}=\sigma (W_{r}\cdot h_{t-1}+b_{r})$$ (15)

The gate unit dynamically adjusts the image feature *q*_*t*_ with attention information, so that the attention mechanism of image feature can fully pay attention to the global and local features in the image fusion features. In this way, attention can be paid to the information in the global and local image fusion features more accurately. The calculation process is as follows:

$$v_{t}=r_{t}⊗q_{t}$$ (16)

Where ⊗ is the matrix multiplication operation.

Through the above calculation process, the dynamically adjusted image fusion feature *v*_*t*_ with attention weight information is obtained. The detailed calculation process of the LSTM network input and output is as follows:

(1) Given the image feature vector *v*_*t*_ and the word vector *w*_*t*_ in the training dataset, the LSTM network input *x*_*t*_ is obtained as follows:
$$x_{t}=\{v_{t};w_{t}\}$$ (17)
  Where, {;} represents the splicing of two vectors.
(2) The semantic hiding state *h*_*t*_ of LSTM at the current time is obtained from the previous value and the fused vector *x*_*t*_, as follows:

$$h_{t}=LSTM(x_{t},h_{t-1})$$ (18)

(3) Finally, a SoftMax function is applied to the output of the fully connected layer as follows:
$$p_{t}=\tanh(W_{p}h_{t}+b_{p})$$ (19)
$$y_{t}=\mathrm{softmax}(W_{y}p_{t}+b_{y})$$ (20)
  Where *W*_*p*_, *W*_*y*_ and *b*_*p*_, *b*_*y*_ are the weight parameters and bias parameters that the Multilayer Perceptron needs to learn.

### 3.4 Description statement generation phase

In the model reasoning and testing stage of image description generation, a probability vector is generated where each element represents the probability value of each word vector in the dictionary at the current time. In the description generation stage, a greedy search algorithm is mostly used to find the word vector with the highest probability as the predicted word vector at the current time. Although this algorithm can ensure that each word is optimal by itself, they may be less desired when combined into a sentence.

To improve the efficiency of the search, we use the beam search algorithm based on breadth-first search. It generates all successors of the states at the current level, sorting them in increasing order of heuristic cost. However, it only keeps a predetermined number of optimal nodes while pruning the other nodes. This algorithm can reduce the computational cost and yield sentences that are more fluent.

## 4. Experimental design and result analysis

### 4.1 Dataset and evaluation index

The ICC Chinese image description dataset [21] was used for the experiment. The data set contains 210000 training pictures, 30000 verification pictures and 30000 test pictures. Each picture has five Chinese image description sentences corresponding to it.

In order to conduct a fair comparison between different models, BLEU (1–4) [29], METEOR [30], ROUGEL [31] and CIDEr [32], which are widely used in the field of image content description, are used as evaluation indexes. The CIDEr evaluation index is specially designed for image description task, which can objectively evaluate the performance of image description model.

### 4.2 Data preprocessing and experimental parameter setting

Before the model training, the images of the original dataset are uniformly scaled to 256*256 pixels. In order to increase the generalization ability of the model, the scaled images are randomly cropped to 224*224 pixels and randomly rotated by 15°. Using the "Jieba" word segmentation tool, the description text labels in the dataset are segmented, and the words with frequency greater than 5 are reserved. Each word is represented by a unique number to form a dictionary of the dataset. The final size of the dictionary is 7768.

In the encoder, the IoU threshold of NMS algorithm is set to 0.5, and the top 100 candidate frames with higher prediction probability are selected from the filtered candidate frame set. In the decoder, the word vector input dimension and network output dimension of LSTM network are set to 512, and the hidden layer dimension of image feature attention is set to 512. In the model training phase, the Batch Size of batch training is set to 128, and the initial learning rate of encoder and decoder is 0.0001. The model uses Adam [33] to optimize the parameters. In the back-propagation, the gradient of each round of training is trimmed to prevent the gradient explosion of the model. When the word vector is generated by the model, dropout [34] technology is used to prevent the model from over fitting. The parameter value of dropout is 0.5.

### 4.3 Experimental results and analysis

#### 4.3.1 Model training and performance comparison

In the model encoder network, the global feature extraction module ResNet152 and the local feature extraction module ResNet50 and RPN are initialized with the parameters of the pre-training model; the decoder network parameters are initialized with random parameters. In the initial stage of model training, the decoder network does not have the ability to decode. In order to prevent the large error produced by the decoder, the network parameters of the encoder are fixed in the initial stage. When the evaluation index score of the model in the verification set converges, the network parameters of the encoder are fixed. The encoder network and decoder network are trained jointly.

**Fig 6:**
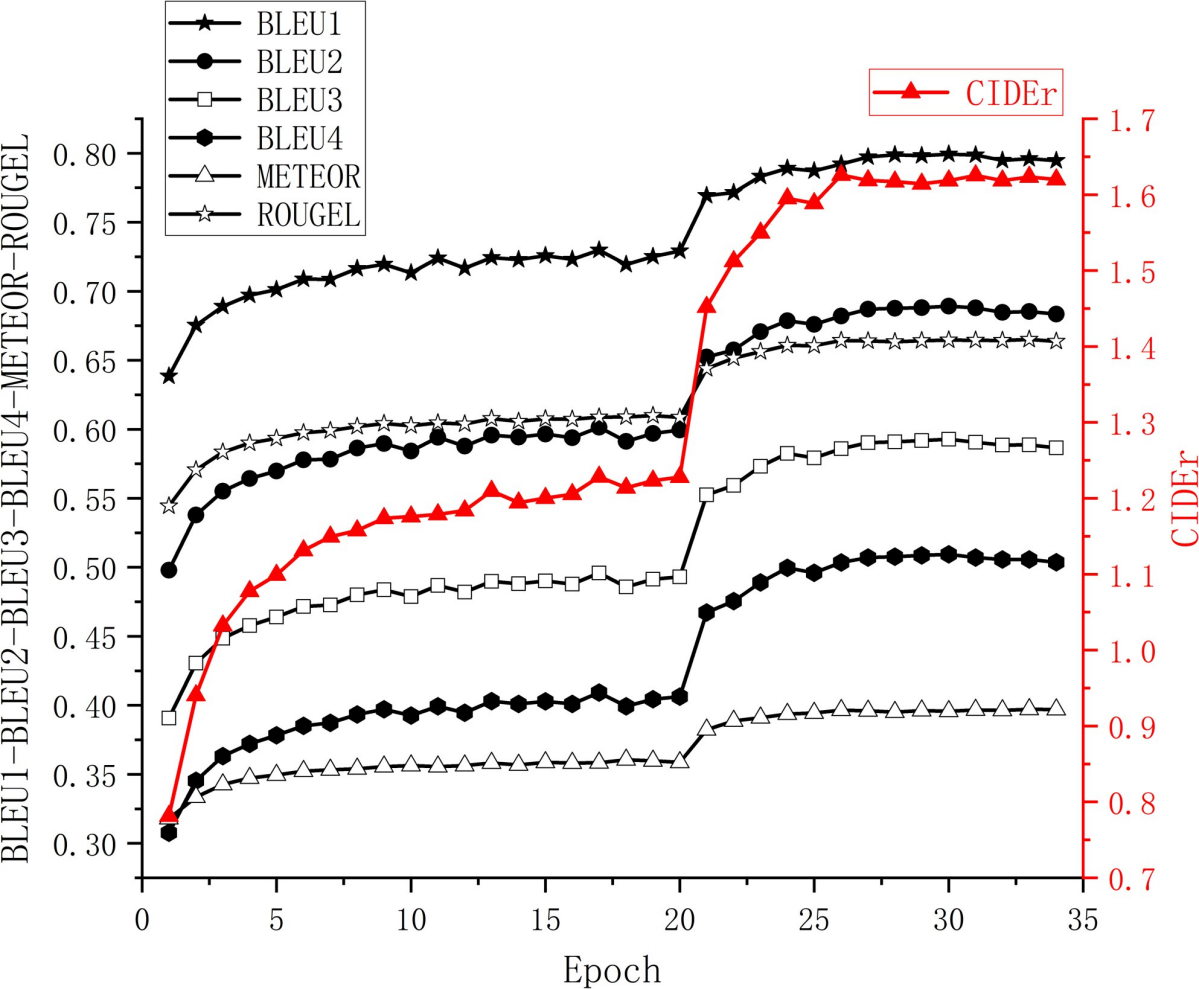
Evaluation score of models in validation set.

The evaluation index scores of the validation set in each epoch of the model training are shown in Fig 6. In order to prevent the random parameters of the decoder network from affecting the pre-training parameters of the encoder, the parameters of the encoder network were frozen in the first 20 epochs. At the 21st epoch, the score of the evaluation index increases significantly because the encoder parameter freeze was removed. Henceforward the coder and decoder of the model were trained jointly, which broke through the bottleneck of the model decoder network, and the evaluation index score of the model has been significantly improved.

In the decoder of Chinese image description method in reference [23], image feature attention mechanism and adaptive attention mechanism are added, and the two attention mechanisms are fused. This method can effectively improve the image understanding ability of the model. In order to verify the influence of the attention fusion mechanism on the global and local image feature fusion mechanism proposed in this research, a comparative experiment as shown in Table 1 is designed with the same dataset.

1. GLF: the model is global and local image feature fusion (GLF) model without attention mechanism.
2. GLF-ATTF: this model is a global and local image feature fusion model with the attention fusion mechanism proposed in reference [23].
3. GLF-ADPATT: this model is a global and local image feature fusion model with a single adaptive attention mechanism.
4. GLF-IFATT: this model is a global and local image feature fusion model with a single image feature attention mechanism.

**Table 1:** Evaluation scores of attention mechanism comparison experiment.

Model	BLEU1	BLEU2	BLEU3	BLEU4	METEOR	ROUGEL	CIDEr
GLF	0.790	0.677	0.579	0.494	0.392	0.657	1.570
GLF-ATTF	0.788	0.674	0.576	0.493	0.394	0.658	1.592
GLF-ADPATT	0.788	0.674	0.575	0.491	0.394	0.658	1.578
GLF-IFATT	**0.798**	**0.687**	**0.591**	**0.507**	**0.397**	**0.665**	**1.624**

According to the experimental results in Table 1, the attention fusion mechanism proposed in reference [23] only improve the performance by a small margin. The score of CIDEr evaluation index only increases by 1.401%, and other evaluation index values are almost the same. In addition, a single adaptive attention mechanism does not improve the performance either. When the single attention mechanism of image features is combined with the global and local image feature fusion method, the scores of all evaluation indices improve compared with those without any attention mechanism, and the score of CIDEr evaluation index increases by 3.439%. Therefore, it can be verified that in the fusion method of global and local image features proposed in this research, the number of local image features is far more than that of global image features. If the adaptive attention mechanism is applied to this model, due to the addition of local detail features, the performance of the model does not improve significantly. The attention mechanism of image feature can calculate the attention information of any image feature according to the word vector, so it has better performance when combined with global and local feature fusion methods.

In order to verify the effectiveness of this model, comparative experiments with other models are carried out using the same dataset, as shown in Table 2.

1. Baseline NIC [21]: this model is the experimental result of NIC model on ICC dataset, and NIC model is often used as the baseline model in this field.
2. BUTD [10]: this model is the Bottom-Up and Top-Down attention model proposed in reference [10], and its experimental results are the reproduction results of reference [11] on ICC dataset.
3. GATT [11]: this model is a global attention model proposed in reference [11], which adds global image features to the BUTD model and uses attention mechanism to improve the performance of the model.
4. G-IFATT [7]: this model uses a single global image feature, and adds the image feature attention [7] to the image Chinese description model, which can reproduce the results on the ICC dataset.

**Table 2:** Evaluation scores of comparison experiment with other models.

model	BLEU1	BLEU2	BLEU3	BLEU4	METEOR	ROUGEL	CIDEr
Baseline-NIC [21]	0.765	0.648	0.547	0.461	0.370	0.633	1.425
BUTD [10]	0.780	0.662	0.561	0.476	0.379	0.637	1.409
GATT [11]	0.782	0.665	0.564	0.477	0.380	0.638	1.433
G-IFATT [7]	0.792	0.670	0.566	0.479	0.382	0.653	1.466
GLF-IFATT	**0.798**	**0.687**	**0.591**	**0.507**	**0.397**	**0.665**	**1.624**

According to the experimental results in Table 2, compared with the Baseline NIC model, the GLF-IFATT model has greatly improved the evaluation indices, and the score of CIDEr evaluation index has increased by 13.96%. Compared with the BUTD model that only uses partial image features, the GLF-IFATT model has a 15.26% improvement in the score of the CIDEr evaluation index. Compared with the GATT model, the GLF-IFATT model yields a 13.33% improvement in the score of the CIDEr evaluation index. Compared with the G-IFATT model, the GLF-IFATT model increases the CIDEr evaluation score by 10.78%. Therefore, the global and local images along with the feature fusion mechanism can effectively improve the image description performance of the model.

#### 4.3.2 Description generation experiment based on beam search

The beam search algorithm is used to optimize the description generation stage of the model. In order to verify the influence of different beam widths on the model evaluation index score, we use the optimal model obtained above and use beam search algorithms with 10 different beam widths to optimize the model description generation stage, and finally use the test set to calculate the evaluation index score of the model.

The evaluation scores of different beam widths are shown in Table 3. It can be seen from the table that when the beam width is 2 and 3, the beam search algorithm has the most obvious optimization effect on the description generation of the model. When the beam width is greater than 3, the evaluation index score of the model begins to increase slowly. When the beam width is 7, the evaluation index score reaches the maximum, and the model achieves the optimal performance.

**Table 3:** Impact of different beam widths on model evaluation scores.

Beam Size	BLEU1	BLEU2	BLEU3	BLEU4	METEOR	ROUGEL	CIDEr
1	0.799	0.688	0.592	0.509	0.395	0.664	1.619
2	0.810	0.705	0.610	0.528	0.398	0.669	1.677
3	0.816	0.712	0.619	0.538	0.400	0.671	1.701
4	0.815	0.713	0.620	0.540	0.399	0.671	1.702
5	0.813	0.711	0.620	0.540	0.399	0.671	1.704
6	0.813	0.711	0.620	0.541	0.398	0.670	1.707
7	0.813	0.712	0.621	0.542	0.399	0.671	1.711
8	0.811	0.709	0.618	0.539	0.397	0.669	1.700
9	0.811	0.710	0.619	0.540	0.397	0.669	1.704
10	0.810	0.710	0.619	0.540	0.397	0.669	1.705

The results with the beam width value of 7 are compared with the Baseline-NIC model and the greedy search algorithm as shown in Table 4.

1) GLF-IFATT-GS: GLF-IFATT model uses greedy search for description generation.
2) GLF-IFATT-BS: GLF-IFATT model uses beam search for description generation.

**Table 4:** Comparison of different search methods in this model.

Model	BLEU1	BLEU2	BLEU3	BLEU4	METEOR	ROUGEL	CIDEr
Baseline-NIC	0.765	0.648	0.547	0.461	0.370	0.633	1.425
GLF-IFATT-GS	0.798	0.687	0.591	0.507	0.397	0.665	1.624
GLF-IFATT-BS	**0.813**	**0.712**	**0.621**	**0.542**	**0.399**	**0.671**	**1.711**

It can be seen that the beam search algorithm, compared with the greedy search algorithm, improves the scores of all evaluation indicators. The BLEU4 evaluation score is increased by 6.90%, and the CIDEr evaluation score is increased by 5.36%. Compared with the Baseline-NIC model, the model optimized by beam search algorithm significantly improved the evaluation index scores. The BLEU4 evaluation score increase by 17.57% and the CIDEr evaluation score increase by 20.07%. Therefore, the beam search algorithm can significantly improve the image description performance of the model.

In addition, the beam search algorithm is applied to the GLF model without the attention mechanism to verify its effectiveness, as shown in Table 5.

**Table 5:** Comparison of different search methods of GLF model.

model	BLEU1	BLEU2	BLEU3	BLEU4	METEOR	ROUGEL	CIDEr
GLF-GS	0.790	0.677	0.579	0.494	0.392	0.657	1.570
GLF-BS	**0.807**	**0.703**	**0.609**	**0.528**	**0.395**	**0.665**	**1.675**

Among them:

1) GLF-GS: GLF model uses greedy search for description generation.
2) GLF-BS: GLF model uses beam search for description generation.

The experimental results show that the beam search algorithm also improves the scores of various evaluation indicators in the GLF model that does not use the attention mechanism. For example, the BLEU4 score increase by 6.88% and the CIDEr score increase by 6.69%.

In addition, a qualitative comparison of the image description sentences generated by the G-IFATT model with only global image features and the GLF-IFATT model with the global and local image feature fusion is shown in Table 6.

**Table 6:** Subjective comparison of description effects.

Image Name*	ae6247fa31198ca5eb727e7ea1b7f733ae882aeb.jpg	00f440a6cb45bd1ced15c1bfad853b99c9d93b7e.jpg	1d393da03723bd3ca9e3c49fed98e6187748ee03.jpg
Image ID*	7750225178903012713	6756237279709039378	5189223105564507622
G-IFATT	大棚里有两个弯着腰的人在采摘草莓	道路上有三个穿着各异的人在骑自行车	室外的空地上有两个戴着头盔的人在工作
	(Two hunched over people picking strawberries in a greenhouse)	(There are three people in different clothes riding bicycles on the road)	(There are two men in helmets working in the open space outside)
GLF-IFATT	大棚里站着一个右手拿着桶的男人	道路上有三个戴着头盔的人在骑自行车	室外有三个戴着安全帽的男人在工作
	(A man with a bucket in his right hand is standing in the greenhouse)	(There were three men in helmets riding bicycles on the road)	(Outside there were three men in hard hats working)

* Images can be found and viewed in the test dataset "AIC-ICC Dataset Test A" by image name and image ID.

In the first example of Table 6, the description generated by the G-IFATT model appears “草莓(strawberries)”, but there is no strawberries in the image, while the GLF-IFATT model accurately describes the details of “右手拿着桶(with a bucket in his right hand)” and correctly describe the number of people in the image. In the second example, the GLF-IFATT model successfully describes the details of "helmet"; In the third example, the G-IFATT model does not correctly describe the number of people in the image, while the GLF-IFATT model accurately describes the details.

## 5. Conclusion

This paper proposes a Chinese description model of image content based on the fusion of global and local image features. Based on the encoder decoder network structure, the model is improved in the encoding phase, the decoding phase and the description generation phase. In the encoding stage, the global and local detail information of the image are extracted respectively, and the extracted features are sent to the feature fusion module to obtain fusion features. In the decoding stage, the attention mechanism of image features is added to pay attention to more important image features, which is specifically effective for the fused image features. In the description generation phase, the beam search algorithm is used to optimize the generation process of Chinese description of the model image. Finally, the effectiveness of the proposed model was verified by comparative experiments.
